# Age-based mating success in the codling moth, *Cydia pomonella*, and the obliquebanded leafroller, *Choristoneura rosaceana*

**DOI:** 10.1673/031.013.14701

**Published:** 2013-12-11

**Authors:** Vincent P. Jones, Nik G. Wiman

**Affiliations:** Department of Entomology, Tree Fruit Research and Extension Center, Washington State University, I 100 N. Western Ave., Wenatchee, WA 98801

**Keywords:** population dynamics, sexual senescence, tortricid moths, wind-tunnel

## Abstract

In this study, the passage of spermatophores was examined between 1-day-old males mated in no-choice situations with females 0, 2, 4, or 6 days old and the converse for both the codling moth, *Cydia pomonella* (L.) (Lepidoptera: Tortricidae), and the obliquebanded leafroller, *Choristoneura rosaceana* (Harris). For *C. pomonella*, female age had no effect on the passage of spermatophores from males, and only 6-day-old female *C. rosaceana* had reduced spermatophore number. The ages of moths at the time of mating had a greater effect on males, with *C. pomonella* males older than 2 days showing significant reductions in the ability to successfully pass a spermatophore to 1-day-old females. For *C. rosaceana*, 2- and 6-day-old males were significantly less likely to pass a spermatophore, but reduced transfer from 4-day-old males did not reach statistical significance. Wind-tunnel assays were used to evaluate the ability of 1- or 4-day-old males to fly upwind and successfully contact a young calling female. Four-day-old males were more likely to initiate flight upwind, but were less efficient at finding and contacting the females than younger males. This study suggests that evaluation of multiple components of the mating process are required to understand the effect of age at the time of mating on population dynamics of these moths.

## Introduction

The widespread use of mating disruption for the codling moth, *Cydia pomonella* L. (Lepidoptera: Tortricidae), has lead to an increased interest into how pheromones mechanistically impact population growth. Barclay and Judd (1995) provided a conceptual framework for mating disruption that included behavioral and population growth components but lacked validating data. Vickers (1997) performed a partial life table on *C. pomonella* that experienced a delay in mating, and found that as female age at the time of mating increased, reproduction decreased and sterility increased. Jones et al. (2008), working with both *C. pomonella* and the obliquebanded leafroller,, *Choristoneiira rosaceana* (Harris) (Lepidoptera: Tortricidae), also saw the same trends in increased sterility with both species, although the effect was considerably more pronounced in *C. rosaceana* than in *C. pomonella*.

The increase in sterility with increasing age at the time of mating suggests that several possible mechanisms may be acting. These mechanisms include: (1) age-based mate discrimination; (2) a decrease in the ability of older males to produce viable sperm; (3) a decreased ability of older females to successfully store and/or use the sperm; and (4) a decrease in mating caused by age-based behavioral changes. Thus, there are many ways that the age of male and female moths at the time of mating can affect the fertility of the females, and age-related factors may contribute to increased sterility or have other detrimental effects on population growth potential. The age of moths at the time of mating may have important implications for the understanding of population dynamics, the interpretation of pheromone trap catch data in relation to population biology, and the success of mating disruption. The timing and frequency of moth catches from pheromone traps placed in orchards with mating disruption treatments are often compared to traps from untreated orchards in order to assess the effectiveness of pheromone treatments on the assumption that these data represent mating success. However, with age-related mating effects it is clear that the presence or frequency of males could have little bearing on population recruitment without knowing their age, or the age of calling females in the field.

This study was initiated to determine the importance of age-based performance of males and females of both *C. rosaceana* and *C. pomonella*. It focused on lab assays that quantified success in spermatophore transfer in no-choice mating cages, and used flight tunnels to quantify age-based tendencies of males to fly and successfully contact a female as key components of the mating process.

## Methods and Materials

### No-choice mating tests

The *C. pomonella* used in the experiments were reared on an artificial diet and obtained from the USDA-ARS Yakima Agricultural Research Laboratory, Wapato, WA; the insects were of the non-diapausing strain. Moths were shipped to Washington State University's Tree Fruit Research and Extension Center in Wenatchee, WA, as last instar larvae and pupae in cardboard bands. In the laboratory, the pupae were removed from the bands, sorted by sex, and placed in cages (3 L) for emergence in a temperature cabinet (22° C, RH ≈ 75%) with a 16:8 L:D photoperiod. Cages were examined daily for emergence, and newly emerged moths were allocated to one of two separate experiments. In the first, male age was held constant at 1 day, and female age varied from 0, 2, 4, or 6ays d old at the time of pairing. The second study was a reversal of the first, where female age was held constant at 1 day, and male age varied from 0, 2, 4, or 6 days old at time of pairing. Moth pairs were placed into individual 355 mL screened cages for three days before dissection to see if spermatophores had been successfully passed to the female. Each pair was provided with a honey water solution during the three-day period, which allowed spermatophores to harden in females for easier dissection and ensured that discrimination, when it occurred, was a robust response. Dissection methods were similar to Mantey and White (1975), except that the ventral abdominal cuticle of previously frozen females was sliced longitudinally (with micro scissors), and the bursa copulatrix, which is easily identified by the sclerotized signa, was simply pulled from the female with insect pins. The membrane of the bursa was then cut to remove spermatophores.

The *C. rosaceana* were obtained from the Tree Fruit Research and Extension Center colony, which is reared on an artificial pinto bean diet (Shorey and Hale 1965). Last instar caterpillars were placed in plastic cups (96cm^3^) and allowed to pupate. After pupation, the same handling, processing, and treatments described for *C. pomonella* were applied, except that each mating pair was caged individually in 500 mL screened cages rather than the 334 mL cages.

### Laboratory wind-tunnel tests

Using the same source colonies and methods of obtaining moths of a defined age, the ability of 1- or 4-day-old males to respond, fly to a female, and successfully mate in a windtunnel was evaluated. The wind-tunnel was a Plexiglas structure with an angle iron frame (165 cm length × 57 cm width × 57 cm height). A single fan pulled air through the tunnel at 0.3 m sec^-1^ and exhausted the air outside of the lab. Virgin female *C. rosaceana* and *C. pomonella* (< 2 days from adult emergence) were tethered to a Plexiglas platform near the head (upwind side) of the tunnel in the geometric center from the walls and separated from each other by 10 cm. Because the moths’ pheromone components are unique and do not act interspecifically (El-Sayed 2011), both species were run simultaneously. Two *C. rosaceana* and two *C. pomonella* males, one for each species aged 1 and 4 days, were dusted with yellow and red fluorescent dust (Shannon Luminous Materials, Inc., www.blacklite.com), respectively. Males of like ages and dust colors were paired in mesh release canisters so that their colors could not mix. Males and females were photo-conditioned separately from the pupal stage to match the photoperiod of the wind tunnel room, which lacks windows and was equipped with fluorescent lights with a red filter (15 lux) and a timer to regulate photoperiod (16:8 L:D). Humidity was maintained at 48% or higher, and temperature was maintained at 22° C. Male release canisters were placed on a platform identical to the female platform and were aligned directly in the air stream 100 cm downwind from the females. At dusk (artificial), males were released by removing the lids of their respective canisters. A digital video camera with infrared capabilities was used to film the female platform for the duration of 1.5 hr to determine if females had called during the initial part of the scotophase and to provide in depth information on male and female behavior occurring on the mating platform. Each morning, females were removed from the platform and examined in the dark using a black light source (Mineralight Lamp Model UVG-47, Ultra-Violet Products, Inc., www.uvp.com) to determine whether they had been marked by one of the males. The color of the mark indicated the age of the male that made contact. Females were subsequently frozen and dissected to determine their mating status. All males were carefully removed from the tunnel before the experiment was run again. A total of 156 trials were run with *C. rosaceana* and 129 with *C. pomonella*.

**Table 1.**
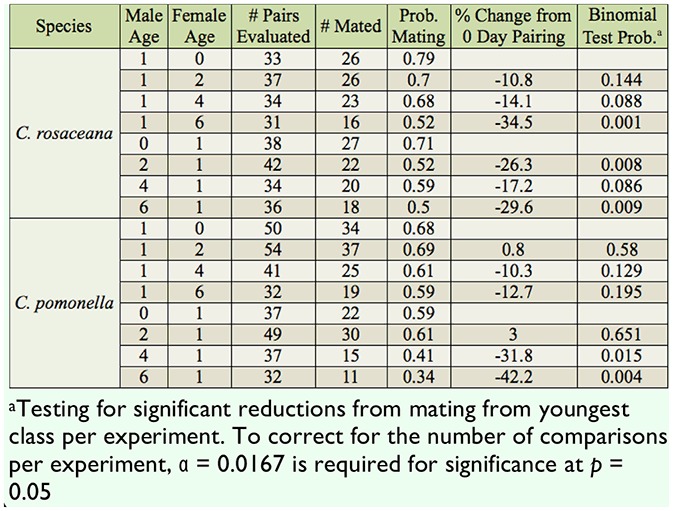
Effect of varying age at time of pairing on mating success in *Choristoneura rosaceana* and *Cydia pomonella* in no-choice pairings.

### Statistical analysis

The goal of the four no-choice experiments was to determine if mating success changed as the age of one sex was held constant and the age of the other varied from 0, 2, 4, or 6 days old. To test this, an exact binomial test statistic (Statacorp, 2010) comparing mating success was used for moths paired on the day of emergence to those of the other ages (2, 4, or 6 days) at time of mating (three 1 *df* comparisons per experiment). To control for the three comparisons per experiment, the Bonferroni inequality was used to ensure that the experiment-wise error rate was α = 0.05 (i.e., α = 0.05/3 = 0.0167 was used to determine the critical value) (Jones 1984). To facilitate comparison between the two species and control for the effect of sex, mating success was standardized across ages by dividing by the success of the 0 age category for each experiment.

The experiments performed in the laboratory wind-tunnel tests were not strictly a choice-type test, because males could have arrived at different times so that females were not presented with an either/or scenario. Instead, the tests addressed three different aspects of behavior under the same conditions: (1) was the proportion of males flying to the platform with the females independent of male age? (2) If the males flew (i.e., took off and oriented toward the platform), was the proportion of males that at least contacted a female independent of male age? (3) Over all the males tested, was the proportion that contacted the female independent of male age?

Because the females were tethered, their ability to reject a male was reduced, and additionally the male's ability to copulate may have been affected by the female's tether. For this reason, mating was not tested statistically for this assay. To analyze the data, 2 × 2 G tests were used to determine if the proportion of the male moths completing the various behaviors (1–3 above) was independent of moth age (SAS Institute 2009).

## Results

### No choice mating tests

As the age of female *C. rosaceana* paired with 1-day-old males increased, the proportion of successful matings decreased compared to females paired on the day of emergence by 10.8%, 14.1%, and 34.5% for females paired 2, 4, and 6, days after emergence, respectively (Table 1). However, the only statistically significant reduction from the females paired on the day of emergence was in the females paired six days after emergence (Table 1).

Instead of the steady decline seen when female *C. rosaceana* age was varied, the mating success of the 2-, 4-, and 6-day-old males decreased by 26.3%, 17.2%, and 29.6%, respectively, when male age was varied compared to the males paired on the day of emergence (Table 1). The reductions were significant for the 2- and 6-day-old females, but fell short of being significant for males paired four days after emergence (Table 1).

When *C. pomonella* female age was varied, mating success changed only slightly (1% increase) compared to females paired on the day of emergence, with a 10% and 13% decrease recorded for females paired 2, 4, or 6 days after emergence. In no cases were there significant differences in mating success compared to the females mated on the day of emergence (Table 1).

The experiments where male *C. pomonella* age was varied showed that there was a 3% increase compared to males paired on the day of emergence, and a 31.8% and 42.2% decrease in mating when males were paired 2, 4, and 6 days after emergence. Males paired at 4 and 6 days after emergence had a significantly lower probability of mating compared to the moths paired at 0 or 2 days after emergence (Table 1).

**Figure 1.**
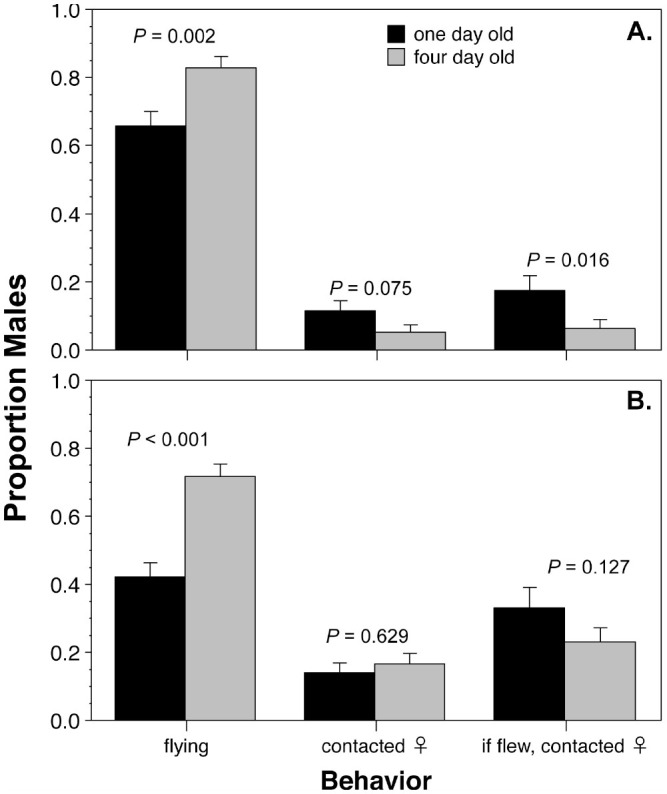
Comparison of the tendency of I - and 4-day-old males to fly and their ability to contact a calling female I 00 cm away in a wind tunnel. A. *Cydia pomonella*. B. *Choristoneura rosaceana*. High quality figures are available online.

### Wind-tunnel tests

In the wind tunnel tests, the 4-day-old males were significantly more likely to respond (by flying upwind to the platform) than 1-day-old males for both species (Figure 1 A, B; *C. pomonella G =* 10.0, 1 df, *p =* 0.002; *C. rosaceana G=* 28.1, 1 df, *p <* 0.001). In *C. pomonella*, 1-day-old males that flew were significantly more likely to contact the female than 4-day-old males (Figure 1A; *G =* 5.8, 1 df, *p =* 0.016). *C. rosaceana* showed the same trend, with 1-day-old males that flew being more likely to contact the female (Figure 1B), but the differences were not statistically significant (G = 2.3, 1 df, *p =* 0.127). In both species, the tendency of older males to fly more frequently was cancelled by the younger males ability to actually contact the female, so that overall there were no significant differences in rates of contact between age categories (Figure 1A, B).

## Discussion

The wind-tunnel experiments showed that older males of both species had a greater tendency to fly than younger males, but that younger males tended to be more efficient at finding females. These two factors cancelled each other out in both species, so that the proportion of younger and older individuals actually contacting the female was similar. This result suggests that, within the time intervals that were tested, flight performance is not likely to be a significant factor in agebased mating success in normal (non-mating disruption) circumstances. In mating disruption circumstances, the effects cannot be directly estimated from these studies, because the balance between flight tendency and searching may be altered by the extreme concentration of pheromone present in the environment. For example, males are clearly excited by the presence of pheromone, and high concentrations of pheromone in mating disruption situations may cause young males to be as active as older males, thus increasing young males ability to mate. However, depending on why younger males in this test were more efficient at finding females (e.g., more persistence in following the pheromone plume versus better perception of the pheromone), the balance could shift in unpredictable ways (Stelinski et al. 2003; Stelinski et al. 2004).

**Figure 2.**
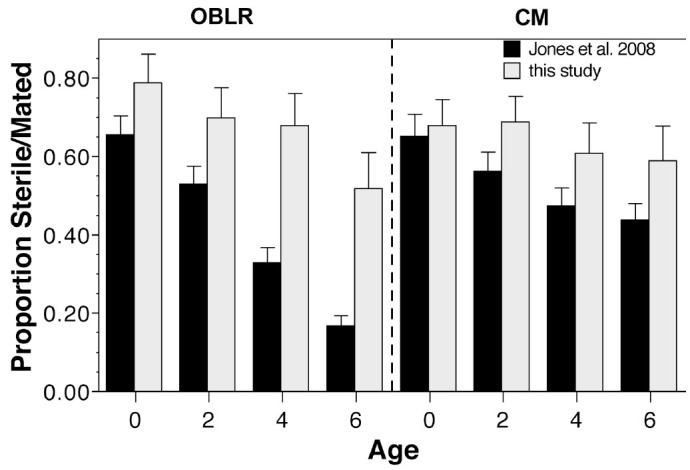
Comparison of the effect of female age for *Cydia pomonella* (CM) and *Choristoneura rosaceana* (OBLR) on the ability to pass a spermatophore (this study) and percentage of mating pairs that were sterile (data from Jones et. al. 2008). Error bars are SEM. High quality figures are available online.

The no-choice tests showed that female age in both species had relatively little to do with the ability of young males to pass a spermatophore; only the 6-day-old *C. rosaceana* females showed significant reductions in spermatophore presence. These results are similar to those reported by Howell (1991), who stated that 4- to 6-day-old virgin *C. pomonella* females mate as readily as younger females. Earlier studies appear to contradict the results of the present study, in that life table data taken in our laboratory using the same methods and conditions showed that delayed female mating resulted in increased sterility in both species as female age increased (Figure 2; Jones et al. 2008). However, the key difference between the two studies is that the no-choice experiments reported herein only examined whether a spermatophore was successfully transferred from a male to a female. In contrast, the study of Jones et al. (2008) was a full life table, and measured sterility as the inability of females to lay fertile eggs. Thus, the current study did not quantify other factors (e.g., transport of the sperm to the spermathecae or release of the sperm to fertilize the eggs) that could influence sterility after the spermatophore was passed. The no-choice studies were more of a behavioral assay to evaluate mating tendency, but required much less time and were simpler to run than the long-term life studies. While the no-choice studies cannot replace the life table studies, they do show at least one additional fact: increased sterility is not related to the ability of older females to mate with younger males (at least within the age range tested herein).

Unlike female age, male age did affect the likelihood of successfully passing a spermatophore in both species, with 4- and 6-day-old *C. pomonella* males showing significant reductions, and the 2- and 6-day-old C. *rosaceana* males showing significant reductions, while the reductions associated with 4-day-old males did not quite reach statistical significance. The male effects may be either a reduced ability of older males to pass spermatophores, females discriminating against older males, or less aggressive male pursuit of females as male age increases. In general, male courtship behavior in both species is stereotypical, but is more complex for females (Castrovillo and Cardé 1980; Curkovic et al. 2006). Female *C. rosaceana* often interrupt copulation attempts by males, and thus may be attempting to select their sexual partners (Curkovic et al. 2006). Male age may be one of the criteria used by females in their mate selection.

In the final analysis, the data showed that single-sex models of the effect of delayed mating are unrealistic. In addition, it is clear that agebased effects are complicated and require examination of several aspects of the matefinding and mating process. No-choice life table studies may give the answer in terms of final age-based sterility and allow the evaluation other factors, such as age-based longevity and fecundity after mating, but they do not address age-based performance in the matefinding process, which can act to either enhance or counteract the tendency for older individuals to be less successful at mating. The need to consider age is particularly important considering the short life-span of both species in field situations, particularly during the heat of the summer (Jones and Wiman 2008) and if unfavorable conditions for mating (high wind velocity or rainfall) occur during key times of the generation.
